# Polystyrene Microplastics Exposure Aggravates Clear Cell Renal Cell Carcinoma Progression via the NF‐κB and TGF‐β Signaling Pathways

**DOI:** 10.1002/advs.202518500

**Published:** 2025-11-27

**Authors:** Shiqi Ye, Jianfeng Xiang, Siqi Zhou, Qintao Ge, Aihetaimujiang Anwaier, Kun Chang, Gang Wei, Jiahe Lu, Xi Tian, Shuxuan Zhu, Yuchen Jiang, Wei Zhang, Tingting Cai, Moran Xu, Dingwei Ye, Danfeng Xu, Tao Wang, Hailiang Zhang, Wenhao Xu

**Affiliations:** ^1^ Department of Urology Fudan University Shanghai Cancer Center Department of Oncology Shanghai Medical College Shanghai Genitourinary Cancer Institute Fudan University Shanghai 200032 China; ^2^ Department of Urology Ruijin Hospital Shanghai Jiao Tong University School of Medicine Shanghai 200025 China; ^3^ Department of Interventional Oncology Renji Hospital Shanghai Jiao Tong University School of Medicine Shanghai 200127 China; ^4^ Department of Urology Huadong Hospital Fudan University Shanghai 200040 China; ^5^ Beijing Key Laboratory of Diabetes Research and Care Department of Endocrinology Beijing Diabetes Institute Beijing Tongren Hospital Capital Medical University Beijing 100730 China

**Keywords:** clear cell renal cell carcinoma (ccRCC), epithelial‐mesenchymal transition (EMT), nuclear factor‐kappa B (NF‐κB), polystyrene microplastics (PS‐MPs), transforming growth factor‐beta (TGF‐β)

## Abstract

Polystyrene microplastics (PS‐MPs) are increasingly associated with carcinogenesis. However, their specific role in clear cell renal cell carcinoma (ccRCC) remains unclear. In this study, the microplastics in ccRCC tissues and normal adjacent tissues (NAT) are detected utilizing Py‐GC/MS, LDIR, and SEM. Tumor functional assays are conducted to assess the effects of PS‐MPs on ccRCC cellular behaviors. Transcriptomic alterations induced by PS‐MPs are characterized via RNA‐sequencing (RNA‐seq) analysis. Key signaling pathways are investigated through immunoblotting, immunocytochemistry, and ELISA. PDO and CDX models are employed to evaluate the effects of PS‐MPs on ccRCC progression and intervention strategies. The results demonstrate that PS‐MPs are markedly abundant in ccRCC tissues compared to NAT. Cytoplasmic accumulation of PS‐MPs promotes malignant phenotypes in ccRCC cells. RNA‐seq analysis demonstrates significant enrichment of oncogenic pathways following PS‐MPs exposure. Mechanistic validation confirms PS‐MPs exposure activates the NF‐κB and TGF‐β pathways in ccRCC. In preclinical models, PS‐MPs accelerate ccRCC growth, which is attenuated by treatment with the pathway inhibitors. In conclusion, this study provides the first comprehensive evidence that PS‐MPs exacerbate ccRCC progression through activating the NF‐κB and TGF‐β pathways. These findings establish PS‐MPs as an environmental risk factor for ccRCC and identify potential therapeutic targets to counteract PS‐MPs‐mediated oncogenic effects.

## Graphical abstract

This research provides the first comprehensive evidence that PS‐MPs exacerbate ccRCC progression by activating the NF‐κB and TGF‐β pathways. These findings establish PS‐MPs as an environmental risk factor for ccRCC progression and identify the NF‐κB and TGF‐β signaling as potential therapeutic targets to mitigate the adverse effects of ‐PS‐MPs exposure.

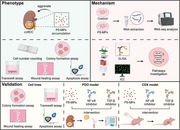

## 1 Introduction

In recent years, plastic pollution has emerged as a critical global environmental issue, raising substantial concerns regarding the impact of microplastics (MPs) on both the ecosystem and human health.^[^ ^1^, ^2^, ^3^ ^]^ MPs are solid plastic fragments and polymeric particles, ranging in size from 1 µm to 5 mm.^[^ ^1^, ^4^, ^5^ ^]^ Due to the widespread misuse and indiscriminate disposal of plastic products, MPs are widespread and mobile in the environment, being detected in water, air, soil, and food.^[^ ^1^, ^6^ ^]^ Humans are exposed to MPs through inhalation, ingestion, and dermal contact.^[^ ^7^, ^8^ ^]^ Consequently, MPs can easily enter and distribute throughout the human body.^[^ ^9^ ^]^ MPs have been detected in human tissues, organs, and bodily fluids.^[^ ^8^, ^10^ ^]^ A growing body of evidence has highlighted the potential association between MPs exposure and numerous diseases such as infertility, diabetes, growth retardation, and cardiovascular diseases.^[^ ^7^, ^11^, ^12^, ^13^, ^14^ ^]^

The relationship between MPs exposure and tumorigenesis has recently garnered increasing scientific interest.^[^ ^15^, ^16^, ^17^ ^]^ Many studies have reported that MPs abundance is significantly higher in tumor tissues compared to normal adjacent tissues (NAT) across diverse malignancies, such as prostate cancer, penile cancer, and colorectal adenocarcinoma.^[^ ^18^, ^19^, ^20^ ^]^ Furthermore, MPs exposure levels have been shown to increase with disease progression in cervical cancer.^[^ ^21^ ^]^ A comprehensive meta‐analysis encompassing 43 studies and 1 006 510 patients corroborates a positive correlation between MPs exposure and elevated cancer risk.^[^ ^22^ ^]^ Experimental studies have demonstrated that MPs accelerate colorectal cancer development in animal models and induce oxaliplatin resistance through modulation of cellular autophagy.^[^ ^23^ ^]^ Exposure to polystyrene‐MPs (PS‐MPs) enhances proliferation, invasion, and migration behaviors in gastric cancer cells and confers resistance to both chemotherapy and monoclonal antibody therapy.^[^ ^24^ ^]^ Similarly, PS‐MPs exposure promotes these tumor characteristics in breast cancer cells.^[^ ^25^, ^26^ ^]^ Polyethylene terephthalate (PET) and polyethylene (PE) have been shown to stimulate hepatocellular carcinoma cell proliferation, and PE‐MPs exposure promotes the proliferation of skin cancer cells.^[^ ^21^, ^27^ ^]^ Despite these indications of carcinogenic potential, the precise mechanisms underlying MPs‐induced oncogenesis remain largely unexplored.

Within the field of nephrology, increasing attention is being directed toward the effects of MPs on renal health.^[^ ^28^ ^]^ Recent studies have demonstrated the presence of MPs in human renal tissues.^[^ ^29^, ^30^ ^]^ Among the MPs identified in ten human kidneys, PE and PS were the most prevalent.^[^ ^29^ ^]^ Our previous work highlighted the synergistic effects between PS‐MPs exposure and a high‐fat diet in reshaping the mouse renal microenvironment by utilizing the single‐cell RNA‐sequencing (scRNA‐seq) approach.^[^ ^31^ ^]^ We demonstrated that PS‐MPs not only promote renal fibrosis and injury, but also upregulate pathways implicated in cancer promotion.^[^ ^31^ ^]^ These findings provide a compelling rationale for investigating the role of PS‐MPs in kidney cancer progression.

Renal cell carcinoma (RCC) ranks among the top ten most common malignancies worldwide in 2024, with a rapidly increasing incidence.^[^ ^32^ ^]^ Clear cell renal cell carcinoma (ccRCC) accounts for ≈80% of all RCC cases and is characterized by its aggressive behavior and poor prognosis at advanced stages.^[^ ^33^ ^]^ Given the accumulation of MPs in the kidneys and our previously observed alterations in the renal microenvironment following PS‐MPs exposure, it is crucial to elucidate whether and how MPs influence ccRCC progression.^[^ ^28^, ^31^ ^]^ A recent study demonstrated the widespread presence of MPs in human ccRCC tissues.^[^ ^34^ ^]^ However, the direct contribution of MPs to ccRCC progression and the specific molecular mechanisms involved remain poorly understood.

In this study, we demonstrate that PS‐MPs are markedly abundant in ccRCC tissues compared to NAT. PS‐MPs that accumulate in the cytoplasm significantly enhance the proliferation, migration, and invasion while inhibiting their apoptosis in vitro. Mechanistically, PS‐MPs exacerbate ccRCC progression through activation of the NF‐κB and TGF‐β signaling pathways. These findings are further validated in patient‐derived organoid (PDO) and cell‐derived xenograft (CDX) mouse models. The pro‐tumorigenic effects of PS‐MPs are effectively attenuated by the NF‐κB and TGF‐β inhibitors, without apparent toxicity. Collectively, our findings unveil a novel oncogenic role and specific mechanism of PS‐MPs in ccRCC progression for the first time, and identify the NF‐κB and TGF‐β pathways as potential therapeutic targets. The study not only underscores the health risks of MPs exposure but also provides a basis for developing targeted strategies to mitigate the adverse impacts of MPs on ccRCC.

## 2 Results

### 2.1 Characterization and Accumulation of PS‐MPs in ccRCC Tissues and Cells

As emerging environmental pollutants, MPs, especially PS‐MPs, have been increasingly implicated in carcinogenesis.^[^ ^18^, ^24^, ^35^ ^]^ Our previous research demonstrated the potential carcinogenicity of 1‐µm PS‐MPs in mouse renal tissue.^[^ ^31^ ^]^ Building upon these findings, we aimed to investigate the impact of PS‐MPs on ccRCC progression and to elucidate the underlying mechanisms.

**Figure 1:**
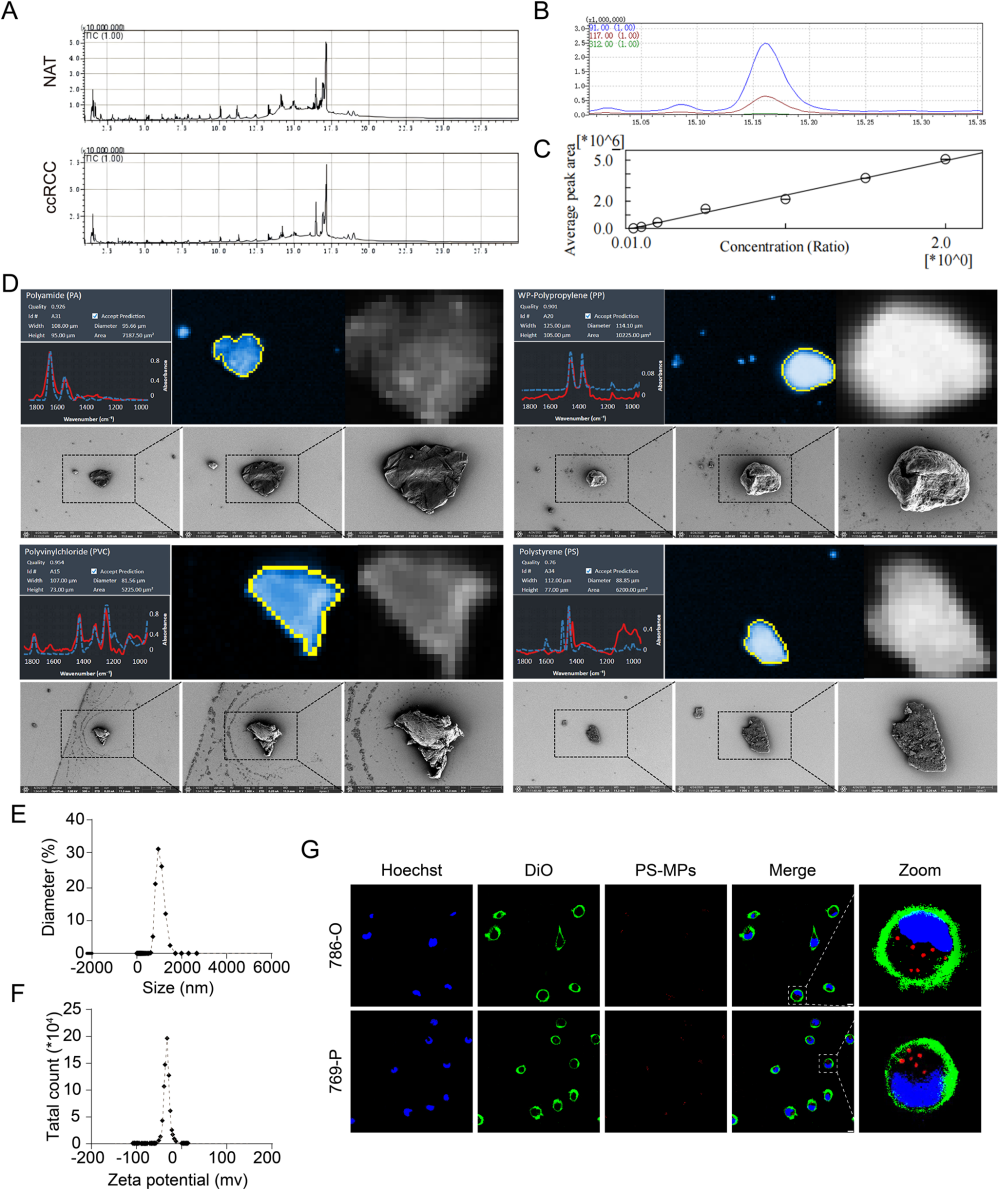
Characterization and accumulation of polystyrene microplastics (PS‐MPs) in clear cell renal cell carcinoma (ccRCC) tissues and cells. A) Total ion current (TIC) profiles from pyrolysis‐gas chromatography‐mass spectrometry (Py‐GC‐MS) analysis of patient‐derived tissues. The upper panel shows normal adjacent tissues (NAT), and the lower panel shows ccRCC tissues. Polystyrene (PS)‐specific peaks are significantly higher in ccRCC tissues. B) Py‐GC‐MS spectrum of PS standard, showing characteristic fragmentation patterns with a prominent ion at m/z 91 for PS identification. C) Quantification of PS‐MPs using the ion intensity at m/z 91, showing a linear relationship between peak area and PS injection amount for accurate quantification. D) Four types of MPs detected by low‐dose ionizing radiation (LDIR) and scanning electron microscopy (SEM) in ccRCC tissues: polyamide (PA), polypropylene (PP), polyvinylchloride (PVC), and PS. E,F) Size distribution (E) and zeta potential (F) analysis of PS‐MPs. G) Intracellular localization of PS‐MPs in 786‐O (upper panel) and 769‐P (lower panel) cells by confocal microscopy. The cells were treated with 10 µg/mL PS‐MPs with a diameter of 1 µm for 72 h. Scale bar is 10 µm. DAPI: blue. DiO: green. PS‐MPs: red.

To assess the presence and types of MPs in ccRCC tissues, we conducted Py‐GC‐MS on paired ccRCC tissues and NAT from three ccRCC patients. As shown in **Figure** **1**A, the total ion current (TIC) profiles revealed distinct differences between NAT and ccRCC tissues. Notably, characteristic PS‐specific fragments were markedly increased in ccRCC, indicating a pronounced enrichment of PS‐MPs within tumor tissues. To validate the identification, PS standards were analyzed by Py‐GC‐MS, showing characteristic fragmentation peaks with a dominant ion at m/z = 91 (Figure 1B). Quantitative calibration revealed a linear correlation between m/z = 91 peak intensity and injection concentration, enabling reliable quantification of PS‐MPs in biological matrices (Figure 1C).

We next performed a comprehensive multi‐polymer analysis, including five common MPs types: PS, PE, polypropylene (PP), polyvinyl chloride (PVC), and polyamide (PA). The quantitative results are summarized in Table S1 (Supporting Information). Across the ccRCC patients, ccRCC tissues exhibited markedly higher concentrations of overall MPs compared to NAT, particularly PS‐MPs. PE and PP were substantially elevated in ccRCC tissues, while PVC and PA were detectable only at low levels in both groups. These results revealed a consistent enrichment pattern of MPs, particularly PS‐MPs, in ccRCC, suggesting that PS‐MPs may be the dominant polymer associated with renal carcinogenesis.

The morphological and compositional identities of representative particles were further verified using LDIR and SEM, confirming the presence of PA, PP, PVC, and PS in ccRCC tissues (Figure 1D). Subsequently, we focused functional analyses on PS‐MPs as the major polymer of interest. Additionally, we investigated whether PS‐MPs could be internalized by ccRCC cells. The characteristics of the applied 1‐µm PS‐MPs were presented in Figure 1E,F. Following treatment of ccRCC cells with 10 µg mL^−1^ of PS‐MPs for 72 h, we observed significant uptake and cytoplasmic accumulation of PS‐MPs (red fluorescent) in ccRCC cells (786‐O and 769‐P) (Figure 1G).

Collectively, these findings provided translational evidence that PS‐MPs, previously shown to promote renal carcinogenic changes in mice, were markedly enriched in human ccRCC tissues and could be internalized by tumor cells, thereby supporting further mechanistic exploration of PS‐MPs‐driven renal tumorigenesis.

### 2.2 PS‐MPs Accelerate the Progression of ccRCC In Vitro

**Figure 2:**
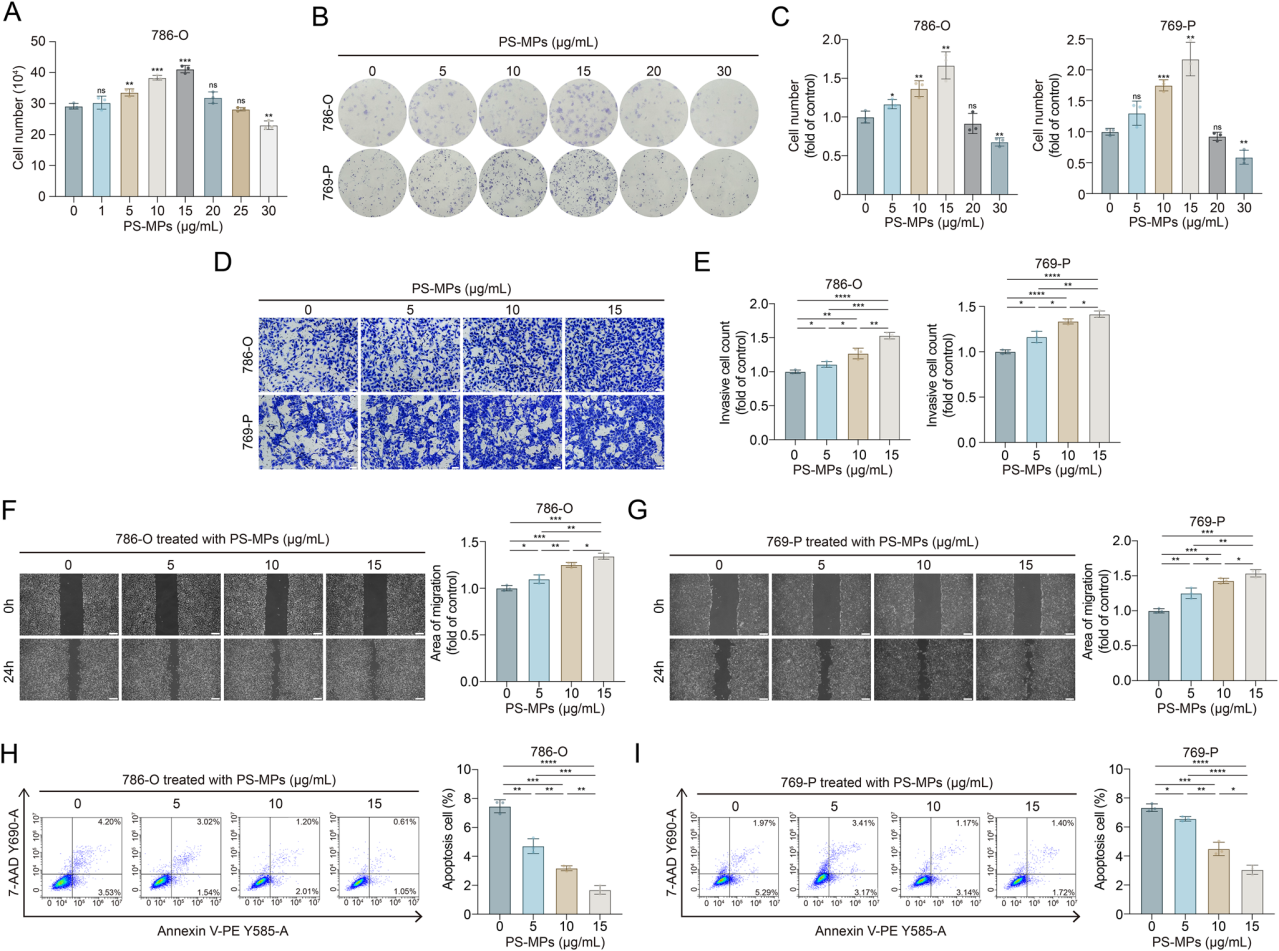
PS‐MPs enhance the malignant biological characteristics of ccRCC in vitro. A) Quantification of 786‐O cell numbers cultured with indicated concentrations of PS‐MPs for 72 h. B,C) Colony formation assay of 786‐O and 769‐P cells cultured with indicated concentrations of PS‐MPs for 72 h. D,E) Transwell assay of 786‐O and 769‐P cells cultured with indicated concentrations of PS‐MPs for 72 h. Scale bar is 100 µm. F,G) Wound healing assay of 786‐O (F) and 769‐P (G) cells cultured with indicated concentrations of PS‐MPs for 72 h. Scale bar is 200 µm. H,I) Flow cytometric apoptosis assay of 786‐O (H) and 769‐P (I) cells cultured with indicated concentrations of PS‐MPs for 72 h. Statistical significance was determined by two‐tailed unpaired *t*‐test (A–C and E–I). ^*^ *p* < 0.05, ^**^ *p* < 0.01, ^***^ *p* < 0.001, ^****^ *p* < 0.0001, and ns *p* ≥ 0.05. Experiments were independently repeated three times with similar results; data from one representative experiment are shown (B, D, and F‐I).

To explore the effects of PS‐MPs on ccRCC, we treated ccRCC cells with increasing concentrations of PS‐MPs. Interestingly, cell proliferation was enhanced with higher PS‐MPs concentrations. Specifically, 15 µg mL^−1^ PS‐MPs exerted the most potent stimulatory effect on ccRCC proliferation, whereas higher PS‐MPs concentrations showed no further enhancement or even inhibitory effects (Figure **2**A). Subsequent colony formation assays corroborated the proliferative effect of PS‐MPs on ccRCC cells (Figure 2B,C). Transwell assays revealed that treatment with PS‐MPs significantly augmented the invasive capability of ccRCC cells (Figure 2D,E). Similarly, wound healing assays demonstrated enhanced migratory capability in PS‐MPs‐treated cells (Figure 2F,G). To further characterize the impact of PS‐MPs on ccRCC cell fate, we assessed apoptosis using flow cytometry, which revealed that PS‐MPs inhibited apoptosis in ccRCC cells (Figure 2H,I).

Collectively, these findings demonstrated that PS‐MPs exacerbated ccRCC progression by promoting proliferation, migration, and invasion while suppressing apoptosis. This highlighted PS‐MPs as an emerging environmental risk factor for ccRCC advancement.

### 2.3 RNA‐Seq Analysis Deciphers the Impact of PS‐MPs on ccRCC Transcriptomic Landscape

**Figure 3:**
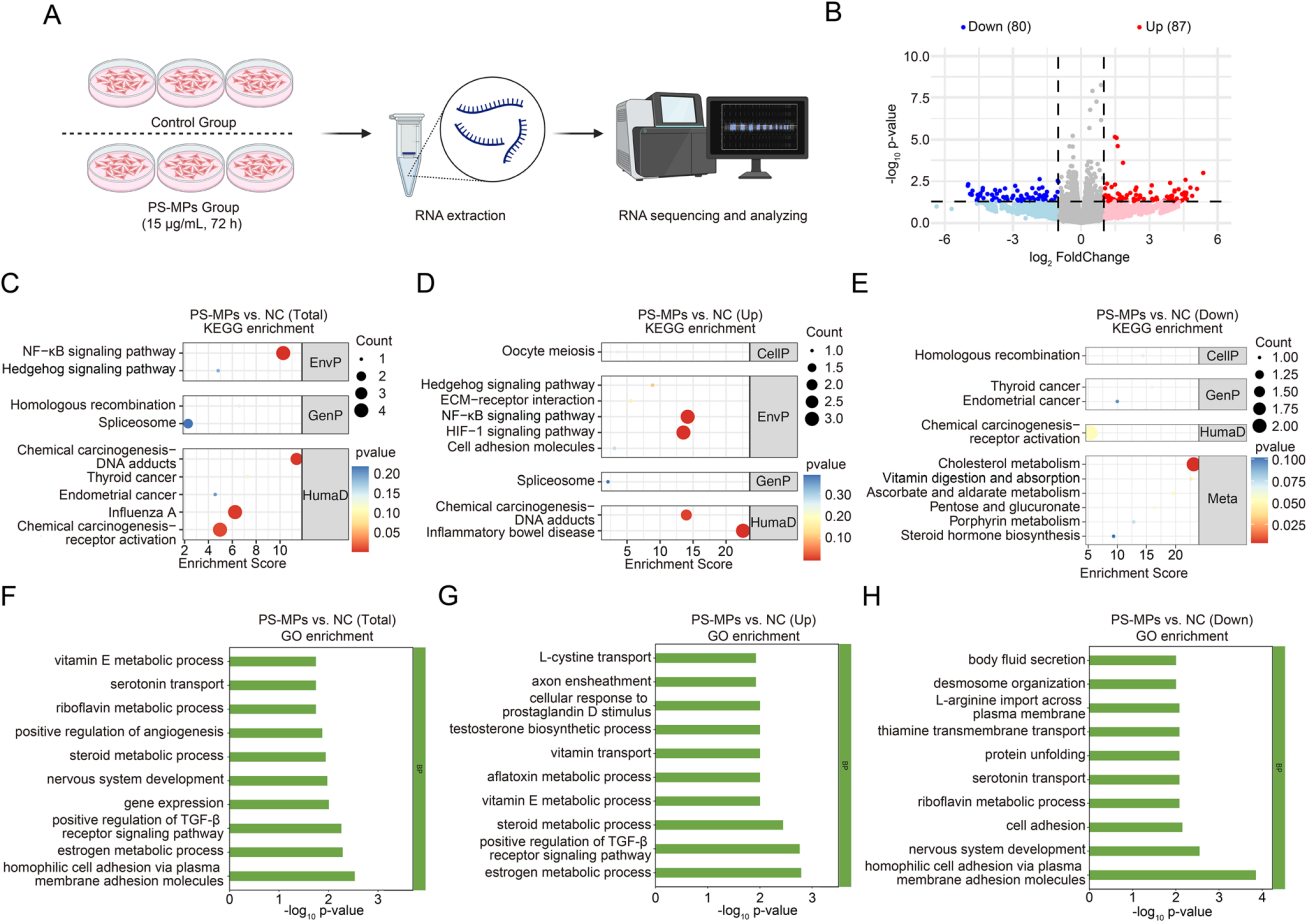
PS‐MPs reshape the transcriptomic landscape of ccRCC cells. A) Schematic illustration for RNA‐sequencing (RNA‐seq) analysis. The PS‐MPs group: 786‐O cells were treated with 15 µg mL^−1^ PS‐MPs for 72 h. B) Volcano plot illustrating differentially expressed genes (DEGs). C–E) The KEGG enrichment analysis of RNA‐seq results. The total enriched pathways (C), up‐regulated enriched pathways (D), and down‐regulated enriched pathways (E) in the PS‐MPs group versus the control group were presented. F–H) The GO enrichment analysis of RNA‐seq results. The total enriched pathways (F), up‐regulated enriched pathways (G), and down‐regulated enriched pathways (H) in the PS‐MPs group versus the control group were presented.

To gain deeper insights into the effects of PS‐MPs on ccRCC, we performed RNA‐seq analysis on ccRCC cells treated with PS‐MPs (**Figure** **3**A). Following exposure to 15 µg mL^−1^ PS‐MPs for 72 h, significant alterations in gene expression profiles were observed in ccRCC cells, with 87 upregulated and 80 downregulated DEGs (Figure 3B). KEGG enrichment analysis highlighted that PS‐MPs upregulated the DNA adducts‐related chemical carcinogenesis pathway in ccRCC (Figure 3C,D). Additionally, the NF‐κB signaling pathway, a classic oncogenic pathway, was notably activated following PS‐MPs treatment (Figure 3C,D).^[^ ^36^, ^37^ ^]^ Conversely, cholesterol metabolism, vitamin digestion and absorption, and ascorbate and aldarate metabolism pathways were downregulated by PS‐MPs treatment (Figure 3E). GO enrichment analysis revealed that PS‐MPs elevated multiple metabolic processes in ccRCC, including estrogen metabolism, steroid metabolism, and vitamin E metabolism (Figure 3F,G). Notably, PS‐MPs activated the TGF‐β pathway in ccRCC cells (Figure 3F,G). However, pathways involved in cell adhesion, protein unfolding, and thiamine transmembrane transport were suppressed by PS‐MPs treatment (Figure 3H). In summary, PS‐MPs reshape the transcriptomic landscape of ccRCC cells and potentially activate critical oncogenic pathways.

### 2.4 PS‐MPs Activate the NF‐κB and TGF‐β Pathways in ccRCC Cells

**Figure 4:**
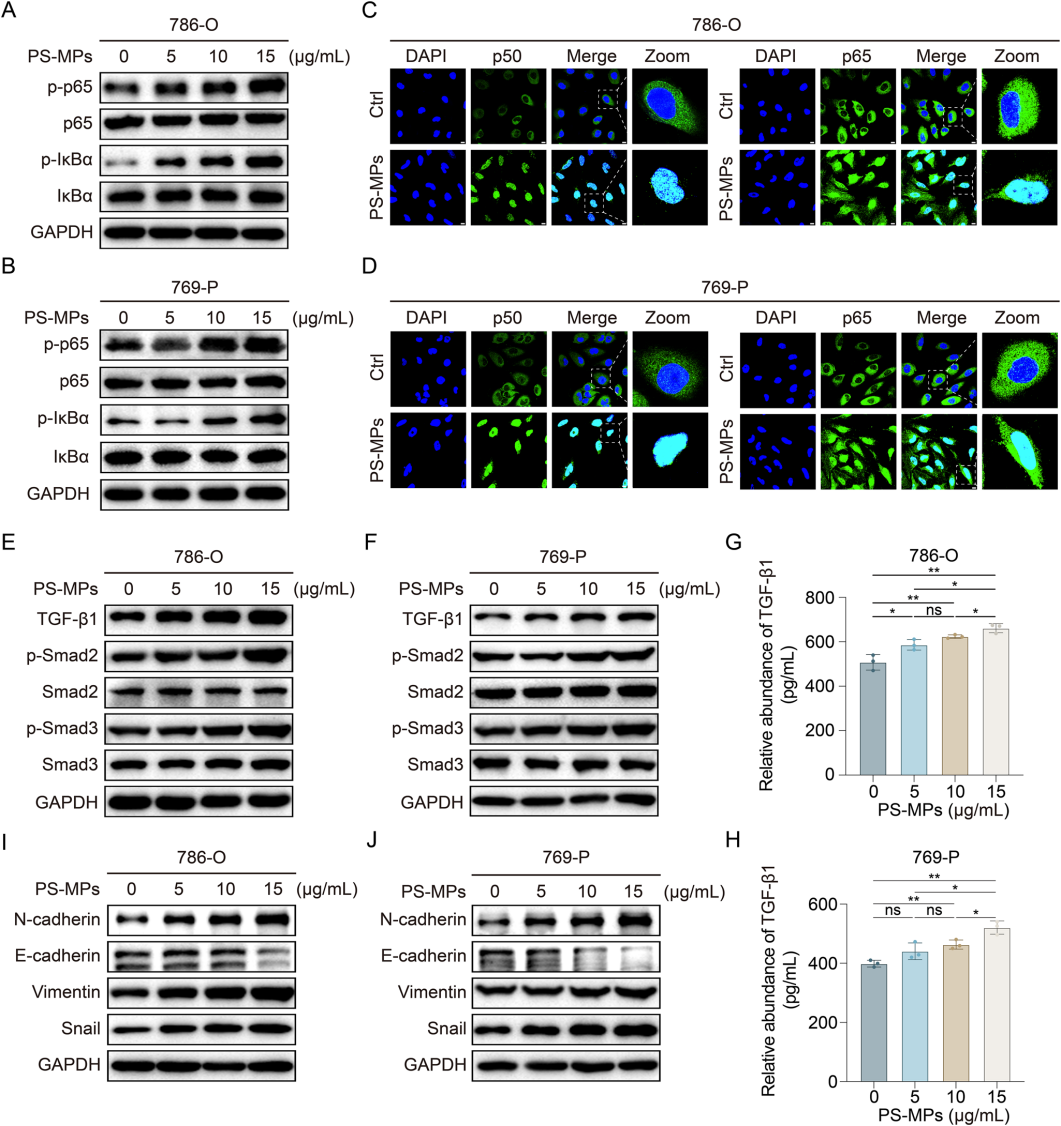
PS‐MPs activate the NF‐κB and TGF‐β pathways in ccRCC cells. A,B) 786‐O (A) and 769‐P (B) cells were treated with indicated concentrations of PS‐MPs for 72 h, and cell lysates were immunoblotted with the indicated antibodies. C,D) Intracellular localization of p50 (left panel) and p65 (right panel) in 786‐O (C) and 769‐P (D) cells treated with or without 15 µg mL^−1^ PS‐MPs for 72 h. Scale bar is 10 µm. E,F) 786‐O (E) and 769‐P (F) cells were treated with indicated concentrations of PS‐MPs for 72 h, and cell lysates were immunoblotted with the indicated antibodies. G,H) Enzyme‐linked immunosorbent assay of TGF‐β1 in 786‐O (G) and 769‐P (H) cells treated with indicated concentrations of PS‐MPs for 72 h. I,J) 786‐O (I) and 769‐P (J) cells were treated with indicated concentrations of PS‐MPs for 72 h, and cell lysates were immunoblotted with the indicated antibodies. Statistical significance was determined by two‐tailed unpaired *t*‐test (G and H). ^*^ *p* < 0.05, ^**^ *p* < 0.01, ^***^ *p* < 0.001, ^****^ *p* < 0.0001, and ns *p* ≥ 0.05. Experiments were independently repeated three times with similar results; data of one representative experiment are shown (A–F and I,J).

To further elucidate the molecular mechanisms driving PS‐MPs‐induced ccRCC progression, we validated the key findings from RNA‐seq analysis. Immunoblotting analysis showed that PS‐MPs exposure induced phosphorylation of p65 and IκBα proteins (**Figure** **4**A,B). Furthermore, ICC assays revealed nuclear translocation of the NF‐κB subunits p50 and p65 following PS‐MPs treatment (Figure 4C,D). These results demonstrated that PS‐MPs activated the NF‐κB pathway.

Concurrently, we investigated the involvement of the TGF‐β signaling pathway, a well‐established inducer of epithelial‐mesenchymal transition (EMT) and tumor progression and metastasis.^[^ ^38^, ^39^ ^]^ PS‐MPs treatment significantly upregulated TGF‐β1 expression and increased the phosphorylation of Smad2 and Smad3 proteins, key downstream effectors of the TGF‐β pathway (Figure 4E,F). ELISA further confirmed elevated TGF‐β1 secretion in the culture supernatants of PS‐MPs‐treated ccRCC cells (Figure 4G,H). These findings indicated that PS‐MPs activated the TGF‐β pathway. Additionally, the EMT process was enhanced by PS‐MPs exposure (Figure 4I,J). Collectively, our findings demonstrated that PS‐MPs activated both the NF‐κB and TGF‐β pathways in ccRCC cells. These results were consistent with the RNA‐seq data and established a molecular framework linking PS‐MPs exposure to oncogenic signaling pathways.

### 2.5 PS‐MPs Drive ccRCC Aggressiveness through the NF‐κB and TGF‐β Pathways

**Figure 5:**
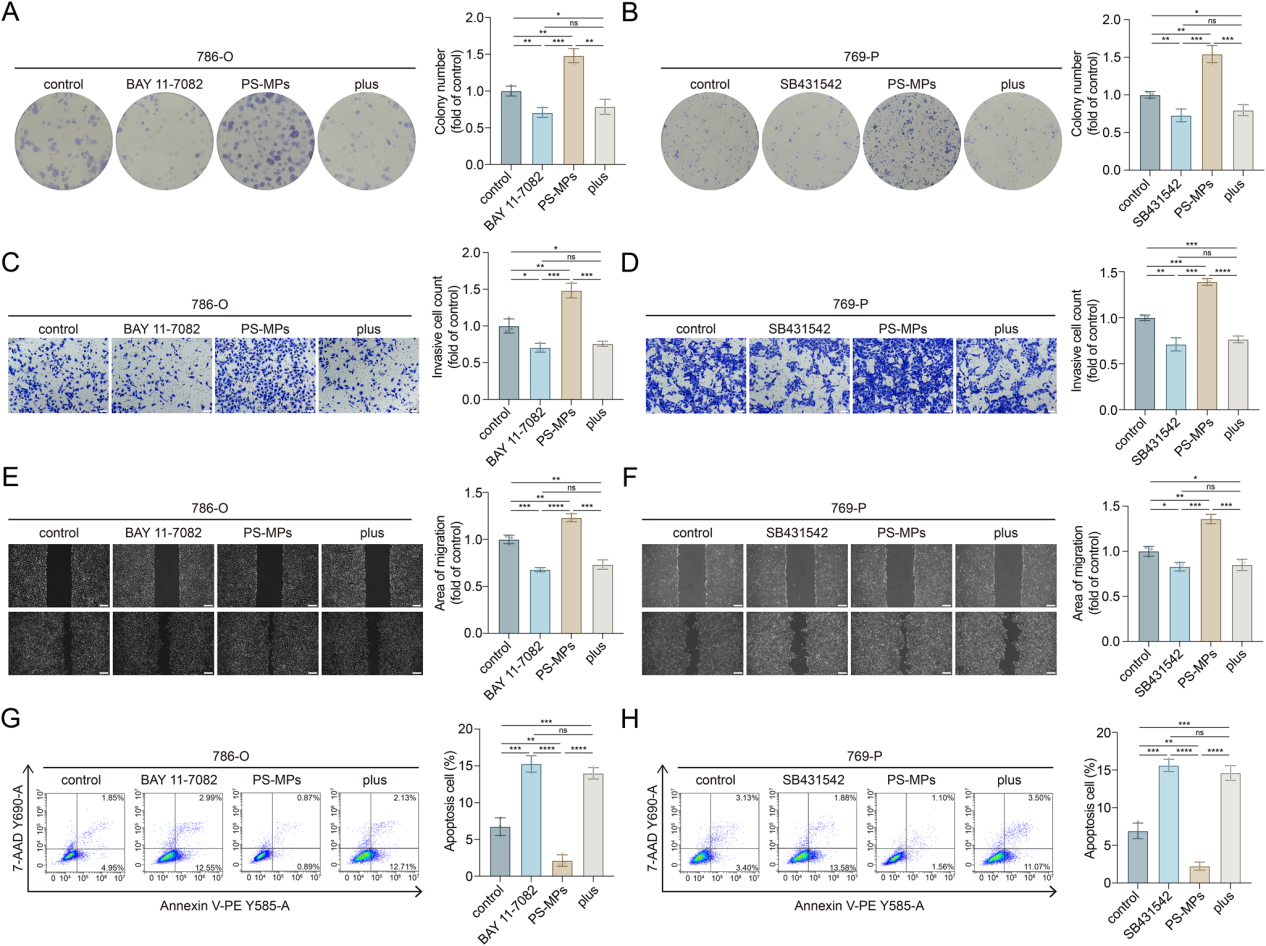
PS‐MPs promote ccRCC progression via the NF‐κB and TGF‐β pathways. A,B) Colony formation assay of 786‐O (A) and 769‐P (B) cells cultured with the indicated treatment. BAY 11‐7082 group: 1 µm BAY 11‐7082 for 72 h. SB431542 group: 1 µm SB431542 for 72 h. PS‐MPs group: 15 µg mL^−1^ PS‐MPs for 72 h. Plus group: BAY 11‐7082 (A) or SB431542 (B) co‐treatment with PS‐MPs for 72 h. C,D) Transwell assay of 786‐O (C) and 769‐P (D) cells cultured with the indicated treatment. Scale bar is 100 µm. E,F) Wound healing assay of 786‐O (E) and 769‐P (F) cells cultured with the indicated treatment. Scale bar is 200 µm. G,H) Flow cytometric apoptosis assay of 786‐O (G) and 769‐P (H) cells cultured with the indicated treatment. Statistical significance was determined by two‐tailed unpaired *t*‐test (A–H). ^*^ *p* < 0.05, ^**^ *p* < 0.01, ^***^ *p* < 0.001, ^****^ *p* < 0.0001, and ns *p* ≥ 0.05. Experiments were independently repeated three times with similar results; data from one representative experiment are shown (A–H).

Building on the above findings, we hypothesized that the activation of the NF‐κB and TGF‐β pathways mediated the pro‐tumorigenic effects of PS‐MPs in ccRCC. To test this, we investigated the impact of specific pathway inhibitors on PS‐MPs‐induced ccRCC progression. Colony formation assays demonstrated that BAY 11‐7082, the NF‐κB pathway inhibitor, effectively reversed the growth‐promoting effects of PS‐MPs (**Figure** **5**A). Similarly, SB431542, the TGF‐β pathway inhibitor, significantly suppressed PS‐MPs‐induced ccRCC proliferation (Figure 5B). These results confirmed the key role of both pathways in PS‐MPs‐mediated tumor growth.

Subsequently, we assessed the role of these pathways in facilitating PS‐MPs‐driven invasion and migration. Transwell and wound healing assays demonstrated that inhibiting either the NF‐κB or TGF‐β pathway attenuated the invasion‐ and migration‐promoting effects of PS‐MPs on ccRCC cells (Figure 5C–F). Additionally, flow cytometry analysis indicated that PS‐MPs failed to suppress apoptosis in ccRCC cells when either the NF‐κB or TGF‐β signaling was inhibited (Figure 5G,H). Taken together, our results demonstrated that PS‐MPs promoted ccRCC progression by activating the NF‐κB and TGF‐β pathways, underscoring their central roles in mediating the oncogenic impact of PS‐MPs exposure in ccRCC.

### 2.6 PS‐MPs Exposure Aggravates ccRCC Progression in Preclinical Models

**Figure 6:**
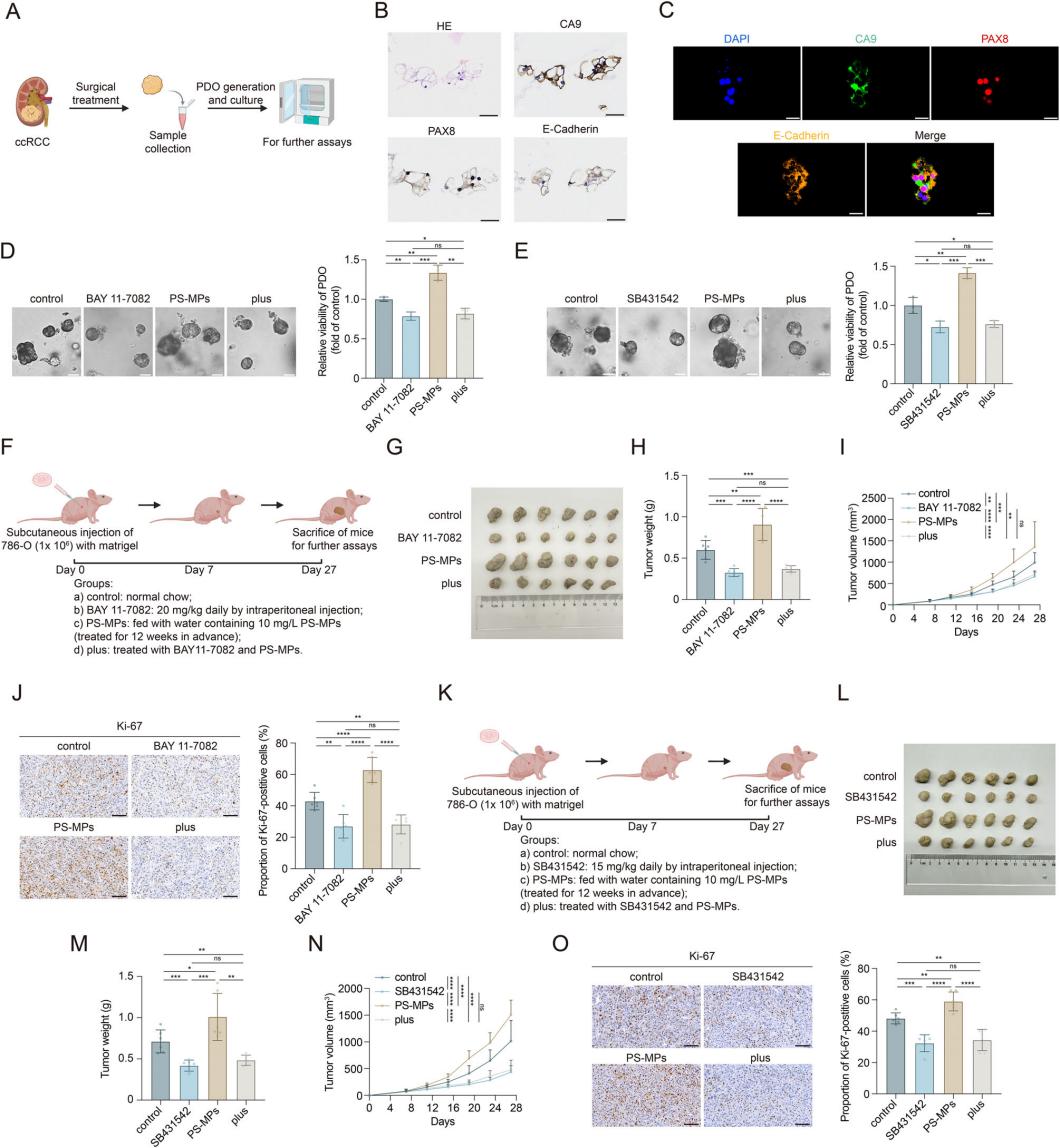
PS‐MPs exposure accelerates tumor progression in preclinical ccRCC models. A) Schematic illustration for the generation of patient‐derived organoid (PDO). B) Representative hematoxylin‐eosin (H&E) and immunohistochemistry (IHC) staining for ccRCC PDO. Scale bar is 30 µm. C) Representative multiplex immunohistochemistry (mIHC) staining for ccRCC PDO. Scale bar is 20 µm. D,E) Representative microscopy image of PDO (left panel) and relative PDO viability (right panel) at the end of the indicated treatment detected by CellTiter‐Glo 3D Cell Viability Assay kit. Scale bar is 50 µm. BAY 11‐7082 group: 1 µM BAY 11‐7082 for 72 h. SB431542 group: 1 µm SB431542 for 72 h. PS‐MPs group: 15 µg mL^−1^ PS‐MPs for 72 h. Plus group: BAY 11‐7082 (D) or SB431542 (E) combined with PS‐MPs for 72 h. F,K) Schematic illustration for the generation of 786‐O cell‐derived xenograft (CDX). BAY 11‐7082 group: 20 mg kg^−1^ BAY 11‐7082 daily by intraperitoneal injection. SB431542 group: 15 mg kg^−1^ SB431542 daily by intraperitoneal injection. PS‐MPs group: fed with water containing 10 mg L^−1^ PS‐MPs (treated for 12 weeks in advance). Plus group: BAY 11‐7082 or SB431542 combined with PS‐MPs. G,L) Tumor images of indicated CDX groups. H,M) Tumor weight of indicated CDX groups. I,N) Growth curves of indicated CDX groups. J,O) Representative IHC staining of Ki‐67 in the indicated CDX groups. Scale bar is 100 µm. Statistical significance was determined by two‐tailed unpaired *t*‐test (D, E, H, J, M, and O), and two‐way ANOVA (I and N). ^*^ *p* < 0.05, ^**^ *p* < 0.01, ^***^ *p* < 0.001, ^****^ *p* < 0.0001, and ns *p* ≥ 0.05. Experiments were independently repeated three times with similar results; data from one representative experiment are shown (D, E, J, and O).

**Figure 7:**
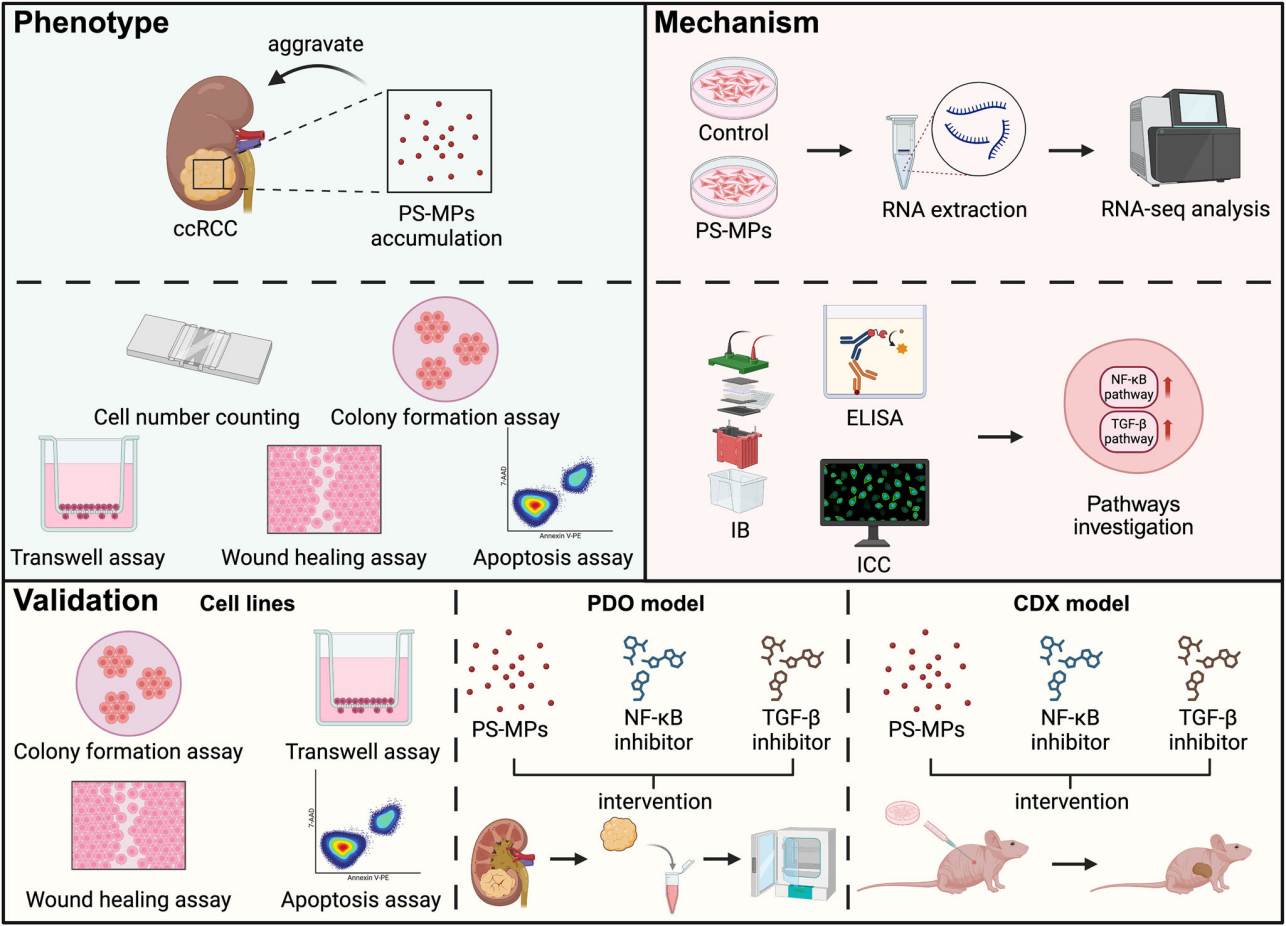
Schematic illustration of the research. This study provides the first comprehensive evidence that PS‐MPs exacerbate ccRCC progression by activating the NF‐κB and TGF‐β pathways. The findings establish PS‐MPs as an environmental risk factor for ccRCC and identify NF‐κB and TGF‐β signaling as potential therapeutic targets to mitigate the adverse effects of PS‐MPs exposure.

To further validate the oncogenic effects of PS‐MPs, we extended our investigations to preclinical models, including PDO and CDX models. We first established a PDO model using surgical ccRCC tissue obtained from a patient (**Figure** **6**A). The PDO was successfully validated through H&E, IHC, and mIHC staining, confirming preservation of key histopathological and molecular features of ccRCC (Figure 6B,C). PS‐MPs treatment significantly enhanced PDO growth, mirroring the pro‐tumorigenic effects observed in vitro. Importantly, the addition of BAY 11‐7082 ‐ or SB431542 ‐ effectively counteracted the growth‐promoting effects of PS‐MPs on ccRCC PDO (Figure 6D,E). To verify these results in vivo, we generated a CDX model by subcutaneously implanting human ccRCC cells (786‐O) into mice (Figure 6F,K). Consistent with in vitro observations, PS‐MPs significantly enhanced ccRCC tumor growth in vivo, and both the NF‐κB and TGF‐β pathway inhibitors mitigated these effects (Figure 6G–J,L–O). Additionally, intraperitoneal administration of these inhibitors showed no apparent toxic effects on the kidneys and livers (Figure S1, Supporting Information). Overall, our preclinical studies demonstrated that PS‐MPs promoted ccRCC progression by activating the NF‐κB and TGF‐β pathways. These findings provided multilevel evidence that PS‐MPs exposure exacerbated ccRCC growth via oncogenic pathways and highlighted potential therapeutic strategies to mitigate their detrimental effects. The schematic illustration of the research is displayed in Figure 7.

## 3 Discussion

The potential health impacts of MPs have garnered considerable scientific interest, with mounting evidence linking MPs exposure to carcinogenesis.^[^ ^40^, ^41^, ^42^ ^]^ However, detailed mechanistic exploration and validation remain limited.^[^ ^40^, ^41^, ^42^ ^]^ To our knowledge, this study represents the first comprehensive investigation into the mechanisms through which MPs influence ccRCC progression, utilizing integrated in vitro and in vivo models.

In this study, our findings demonstrate that PS‐MPs are markedly abundant in ccRCC tissues compared to NAT. Importantly, we show that cytoplasmic accumulation of PS‐MPs promotes ccRCC progression via activation of the NF‐κB and TGF‐β pathways. Furthermore, pharmacological inhibition of these pathways effectively attenuates the pro‐tumorigenic effects induced by PS‐MPs. These findings offer novel insights into how PS‐MPs drive ccRCC malignancy and propose potential therapeutic approaches to counteract PS‐MPs‐mediated adverse effects in ccRCC.

This study specifically focuses on investigating the influence of 1‐µm PS‐MPs on ccRCC for the following reasons. First, several studies have identified PS‐MPs as one of the most prevalent types of MPs detected in both renal tissues and ccRCC tissues.^[^ ^29^, ^34^ ^]^ Second, our previous work utilizing the scRNA‐seq analysis reveals that 1‐µm PS‐MPs exhibit carcinogenic potential in mice kidneys.^[^ ^31^ ^]^ Third, our current study corroborates that PS‐MPs are more abundant in ccRCC tissues than in NAT. Collectively, these considerations motivate our focused investigation into the role of 1‐µm PS‐MPs in ccRCC pathogenesis. Nevertheless, it is important to acknowledge that environmental MPs are highly heterogeneous in their physical and chemical characteristics, such as chemical composition (e.g., PE, PET, PP, PS), particle size (ranging from 1 µm to 5 mm), and morphological features (e.g., spherical, fibrous, irregular fragments).^[^ ^43^, ^44^ ^]^ Emerging evidence indicates that these properties differentially modulate tumorigenic behaviors and molecular mechanisms.^[^ ^43^, ^44^ ^]^ Therefore, while this study provides insights into the effects of 1‐µm PS‐MPs, future research should explore a broader spectrum of particle properties—to more comprehensively assess the health risks posed by MPs exposure in ccRCC and other malignancies.^[^ ^40^, ^41^, ^42^ ^]^

In this study, we demonstrate a concentration‐dependent bidirectional effect of PS‐MPs on ccRCC proliferation in vitro. The RNA‐seq analysis and mechanism investigation indicate that 15 µg mL^−1^ PS‐MPs promote ccRCC proliferation through activation of the NF‐κB and TGF‐β pathways. The pro‐tumorigenic effects of PS‐MPs in ccRCC are consistent with previous reports in the majority of malignancies. For example, PS‐MPs have been reported to accelerate proliferation, invasion, and migration in gastric cancer and breast cancer.^[^ ^24^, ^25^, ^26^ ^]^ In contrast, a higher concentration of PS‐MPs (30 µg mL^−1^) suppresses ccRCC cell proliferation in our study. This inhibitory effect may be attributed to several factors, including increased cytotoxic stress, induction of oxidative damage, and excessive ROS generation.^[^ ^15^, ^17^, ^45^, ^46^ ^]^ Similar dose‐dependent effects have been reported with polystyrene nanoplastics (PS‐NPs), which inhibited proliferation and migration in ovarian cancer cells by altering the tumor microenvironment.^[^ ^47^ ^]^ Further investigations are warranted to elucidate the precise mechanisms of switching PS‐MPs from pro‐tumorigenic to anti‐tumorigenic agents at higher concentrations in ccRCC.

In summary, this preclinical study demonstrates that PS‐MPs exposure accelerates ccRCC progression via activation of the NF‐κB and TGF‐β pathways using in vitro and in vivo models. The findings highlight the therapeutic potential of targeting these signaling pathways to counteract the oncogenic effects of PS‐MPs in ccRCC. Our work contributes to a deeper understanding of the mechanisms by which environmental pollutants influence cancer pathogenesis and highlights novel targets for intervention.

## 4 Conclusion

In conclusion, this study provides the first evidence that PS‐MPs exacerbate ccRCC progression through activation of the NF‐κB and TGF‐β signaling pathways, as demonstrated using in vitro and in vivo models. These findings not only underscore the tumor‐promoting role of PS‐MPs exposure but also propose potential therapeutic strategies to mitigate the risk posed by PS‐MPs exposure in ccRCC (**Figure** **7**).

## 5 Experimental Section

### Study Cohort and Tissue Collection

The patient cohort was selected from individuals diagnosed with ccRCC who underwent surgical resection at the Department of Urology, Fudan University Shanghai Cancer Center (FUSCC) from May to July 2025. The inclusion criteria were as follows: i) histologically confirmed ccRCC diagnosis; ii) no prior chemotherapy, radiotherapy, or systemic therapy; iii) age between 18 and 75 years; iv) Eastern Cooperative Oncology Group (ECOG) performance status of 0 or 1; v) written informed consent obtained for participation in the study. Patients with other active malignancies, autoimmune diseases, or contraindications to surgery were excluded from the study. A total of 6 pairs of fresh tumor and adjacent non‐tumor renal tissues were collected from the patients undergoing partial or radical nephrectomy. The non‐tumor tissues were located at least 2 cm away from the tumor site and were histologically confirmed to be normal renal parenchyma. All tissues were collected immediately after resection, ensuring that the cold ischemia time did not exceed 30 min. The tissues were then snap‐frozen in liquid nitrogen and stored at −80 °C until further analysis.

### Ethics Approval and Consent to Participate

This research complies with all relevant ethical regulations and guidelines. The research design and procedures were conducted in accordance with the principles of the Helsinki Declaration II. The ethics approval of the Urology and Pathology departments and the informed consent from patients in this study were approved by the Institutional Review Board (IRB) of FUSCC (No: 050432‐4‐2108*, FUSCC, Shanghai, China). All animal procedures were performed following protocols approved by the Ethical Committee of FUSCC and in accordance with guidelines from the Institutional Animal Care and Use Committee (IACUC) of FUSCC (No: FUSCC‐IACUC‐S2022‐0626, FUSCC, Shanghai, China).

### Low‐Dose Ionizing Radiation (LDIR) exposure

After collection, all tissue samples underwent a series of standardized processing procedures to evaluate the impact of PS‐MPs exposure on ccRCC tissues. The collected tissue samples were exposed to controlled low‐dose ionizing radiation (LDIR) to mimic environmental stressors. For LDIR exposure, the tissues were placed in a radiation chamber and irradiated with a dose of 0.1 Gy at a dose rate of 0.5 Gy min^−1^. Post‐irradiation, the tissues were incubated at 37 °C with 5% CO_2_ for 24 h before further analysis. LDIR was employed to study its potential role in enhancing the effects of PS‐MPs on tumor progression.

### Scanning Electron Microscopy (SEM)

To examine the ultrastructural effects of PS‐MPs on the tissue samples, SEM was employed. Prior to imaging, tissues were fixed in 2.5% glutaraldehyde at 4 °C for 2 h and post‐fixed in 1% osmium tetroxide for 1 h. Samples were then dehydrated in a graded ethanol series (50%, 70%, 90%, and 100%), critical‐point dried, and coated with gold for imaging. SEM was used to observe the morphology of the cells in the tissues, especially the microstructural alterations associated with PS‐MPs accumulation in the cytoplasm, and to detect any evidence of physical interactions between PS‐MPs and cancer cells.

### Pyrolysis‐Gas Chromatography‐Mass Spectrometry (Py‐GC/MS)

Py‐GC/MS was employed to detect and quantify PS‐MPs in the tissue samples. Tissues were first ground into fine powder in liquid nitrogen, and ≈1 mg of each sample was placed in a quartz tube for pyrolysis. The samples were then subjected to pyrolysis at 500 °C for 20 s in a pyrolysis unit (Pyroprobe 5150, CDS Analytical, Oxford, PA). The pyrolyzed products were then analyzed by GC‐MS (Agilent 7890B/5977A), and the resulting spectra were analyzed for the presence of biomarkers or novel metabolites indicative of PS‐MP interaction. The method was calibrated using a standard PS‐MP reference sample, ensuring precise quantification of polymer‐related residues in the tissues.

### Cell Lines and Culture Conditions

The human ccRCC cell lines, including 786‐O and 769‐P, were purchased from American Type Culture Collection. The cells were cultured in RPMI‐1640 medium with 10% fetal bovine serum (FBS). The cells were routinely tested to exclude mycoplasma contamination. The cells were incubated in the humidified incubator at 37 °C with 5% CO_2_.

### Chemicals

The PS‐MPs with a diameter of 1 µm were purchased from Tianjin Baseline ChromTech Research Center (7‐1‐0100) in accordance with our previous report.^[^ ^31^ ^]^ The NF‐κB inhibitor BAY 11‐7082 (Selleck Chemicals, S2913) and TGF‐β inhibitor SB431542 (Selleck Chemicals, S1067) were used in this study. The treatment time and concentration were described in the indicated figure legends.

### Cell Growth and Colony Formation Assay

For the cell growth assay, single cells were seeded in 6‐well plates (5 × 10^4^ per well) with 2 mL complete culture medium, and cell numbers were counted with a hemacytometer after 72 h. For the colony formation assay, single cells were seeded in 6‐well plates (1 × 10^3^ per well) with 2 mL complete culture medium, and the medium was changed every 4 days. After 10‐14 days, cells were fixed with 4% paraformaldehyde (PFA) fix solution (Beyotime, P0099) for 30 min and stained with crystal violet solution (Beyotime, C0121) for 30 min.

### Transwell Assay

For the transwell assay, single cells were seeded in the upper chamber of transwell chambers (Corning, 353 097) with 200 µL FBS‐free culture medium, and the lower chamber was filled with 800 µL complete culture medium. The Matrigel (Corning, 354 248) was pre‐coated on the microporous membrane of the upper chamber in advance. After 24 h, cells were fixed with 4% PFA fix solution for 30 min and stained with crystal violet staining solution for 30 min.

### Wound Healing Assay

For the wound healing assay, single cells were seeded in 6‐well plates (2 × 10^5^ per well) with 2 mL complete culture medium. When cells became 100% confluent, 100‐µL pipette tips were used to create the wounds. After 24 h, the gap area was photographed and measured.

### Flow Cytometric Apoptosis Assay

The flow cytometric apoptosis assay was performed to detect the apoptotic effect of PS‐MPs exposure on ccRCC cells. The assay was conducted with the Annexin V‐PE/7‐AAD Apoptosis Kit (Multi Sciences, AP104) following the manufacturer's instructions. The data were analyzed with the CytExpert software (Version 2.4). Each experiment was performed in triplicate, and the percentage of apoptotic cells was calculated.

### RNA‐Sequencing (RNA‐Seq) Analysis

Total RNA was extracted with the TRIzol reagent (Invitrogen, 15596026) according to the manufacturer's protocol. RNA quantification and purity were assessed with the NanoDrop 2000 spectrophotometer (Thermo Scientific, USA). The libraries were constructed on the Ilumina Novaseq 6000 platform, and 150 bp paired‐end reads were generated. Raw reads of fastq format were processed with fastp, and the low‐quality reads were removed to obtain the clean reads, which were mapped to the reference genome with HISAT2.^[^ ^48^, ^49^ ^]^ FPKM of each gene was calculated, and the read count of each gene was obtained by HTSeq‐count.^[^ ^50^, ^51^ ^]^ Differentially expressed genes (DEGs) analysis was performed using DESeq2.^[^ ^52^ ^]^ The *p*‐value < 0.05 and log_2_(foldchange) > 1 or log_2_(foldchange) < −1 were set as the threshold for significantly DEGs. The KEGG and GO pathway enrichment analyses were performed to screen the significantly enriched terms and draw the diagrams using R software (v3.2.0).^[^ ^53^, ^54^ ^]^

### Immunocytochemistry (ICC)

For ICC assay, cells were cultured on 35 mm confocal dishes (Biosharp, BS‐20‐GJM) with indicated treatments. For the PS‐MPs localization assay, the culture medium was removed, and PBS was added to wash the cells twice. Then, DiO (Beyotime, C1038) and Hoechst 33342 (Beyotime, C1025) solutions were added to cells following the manufacturer's instructions. After incubation for 10 min, the solution was removed, and PBS was added to wash the cells twice. For p50 and p65 ICC assay, the cells were fixed with 4% PFA for 30 min, incubated with 0.25% Triton X‐100 (Beyotime, P0096) for 10 min at room temperature, blocked with 5% BSA (Beyotime, ST023) for 1 h at room temperature, and incubated with primary antibodies overnight at 4 °C. Next day, the samples were incubated with fluorescein‐conjugated secondary antibodies for 1 h at room temperature and mounted with DAPI (Beyotime, C1006). The samples were visualized with confocal microscopy (Olympus). For ICC analysis, primary antibodies included p50 (Proteintech, 14220‐1‐AP, 1:200) and p65 (Proteintech, 10745‐1‐AP, 1:50). Secondary antibody was anti‐rabbit IgG (H+L) Alexa Fluor 488 Conjugate (Cell Signaling Technology, 4412, 1:2000).

### Immunoblotting

The cells were washed with PBS and lysed with RIPA Lysis Buffer (Beyotime, P0013C) containing 1% protease and phosphatase inhibitors (Beyotime, P1045) for 30 min at 4 °C. Samples were centrifuged at 12 000 rpm for 15 min at 4 °C. Protein lysates were separated by SDS–PAGE and transferred onto PVDF membranes (Millipore, IPVH00010). All membranes were blocked with 5% skim milk in TBST buffer and incubated with the indicated primary antibodies overnight at 4 °C. The Next day, the membranes were incubated with secondary antibodies for 1 h at room temperature. The signal was visualized with LumiBest ECL reagent (ShareBio, SB‐WB011) and detected by the ChemiDocXRS system. The following primary antibodies were used: GAPDH (Proteintech, 60004‐1‐Ig, 1:50 000), E‐cadherin (Proteintech, 20874‐1‐AP, 1:10 000), Vimentin (ABclonal, A19607, 1:10 000), N‐Cadherin (ABclonal, A19083, 1:1000), Snail (ABclonal, A5544, 1:500), TGF‐β (Proteintech, 21898‐1‐AP, 1:1000), p‐smad2 (ABclonal, AP1342, 1:1000), p‐smad3 (ABclonal, AP0727, 1:500), smad2 (Proteintech, 12570‐1‐AP, 1:2000), smad3 (Proteintech, 66516‐1‐Ig, 1:2000), p65 (Proteintech, 10745‐1‐AP, 1:1000), p‐p65 (Proteintech, 82335‐1‐RR, 1:2000), ІкВа (Proteintech, 10268‐1‐AP, 1:5000), and p‐ІкВа (ABclonal, AP0707, 1:500). Secondary antibodies were anti‐rabbit IgG(H+L) (Proteintech, SA00001‐2, 1:5000) and anti‐mouse IgG(H+L) (Proteintech, SA00001‐1, 1:5000).

### Enzyme‐Linked Immunosorbent Assay (ELISA)

The protein level of TGF‐β in cell supernatant was quantified using a commercial human TGF‐β ELISA kit (Shanghai Jianglai Biotechnology, JL10706) in accordance with the manufacturer's instructions. After co‐culturing cells with PS‐MPs for 72 h, supernatant samples were collected and analyzed. The standard curve was calculated based on the standard sample using the four‐parameter logistic regression model, with the concentration on the x‐axis and the 450 nm absorbance on the y‐axis. The concentration of the unknown sample was calculated by interpolating its absorbance value into this fitted standard curve. All samples and standards were run in duplicate, and three independent biological replicates were performed. The detection limit was 31.25 to 2000 pg mL^−1^. Both inter‐assay and intra‐assay coefficients of variation were below 10%.

### Hematoxylin‐Eosin (H&E) and Immunohistochemistry (IHC) Staining Assay

The H&E staining assay was conducted to evaluate the morphological features of the mouse kidney and liver. The protocol consisted of sequential steps: dewaxing, hematoxylin staining, eosin staining, dehydration, sealing, microscope inspection, and analysis.

The IHC staining was performed as follows: dewaxing to water, antigen repair, blocking endogenous peroxidase, serum closure, primary antibody incubation, secondary antibody incubation, diaminobenzidine solution color development, re‐staining nuclei, dewatering, sealing, microscope inspection, and analysis. For the assessment of tumor growth, Ki‐67 staining was specifically evaluated. The percentage of Ki‐67‐positive tumor cells was determined by visual estimation in at least five randomly selected high‐power fields (HPF, 400x magnification). The index was assessed by two independent pathologists in each tumor sample. The H&E and IHC slides were visualized in SlideViewer software (Version 2.5). The detailed methodology of H&E and IHC staining was as described in previous reports.^[^ ^31^, ^55^ ^]^

For IHC analysis, the primary antibodies included CA‐9 (GeneTech, GT224004), PAX8 (GeneTech, GT210204), E‐Cadherin (GeneTech, GT234807), and Ki‐67 (Proteintech, 27309‐1‐AP). The second antibodies were HRP conjugated Goat Anti‐Rabbit IgG (H+L) (Servicebio, 1:10 000) and HRP conjugated Goat Anti‐Mouse IgG (H+L) (Servicebio, 1:10 000).

### Establishment of Patient‐Derived Organoid (PDO) Model

For PDO model establishment, the fresh human ccRCC specimens were collected in ice‐cold PBS supplemented with 1% penicillin/streptomycin to maintain sterility. Then, tissues were mechanically dissociated into small fragments and enzymatically digested at 37 °C for 2 h in mixed digestion medium.

The digested tissues were centrifuged at 500 g for 5 min, and seeded in pre‐warmed 24‐well culture plate pre‐coated with Matrigel (D1 Medical Technology, D23016‐0010). The PDO was cultured with 500 µL PDO culture medium (D1 Medical Technology, K211M09), and the culture medium was replaced every 72 h. The PDO morphology feature was observed and photographed at the indicated time. The PDO viability was evaluated with CellTiter‐Glo 3D Cell viability assay kit (Promega, G9681) following the manufacturer's protocol.

For HE, IHC, and multiplex immunohistochemistry (mIHC) staining, PDO was fixed in pre‐warmed 4% PFA at 37 °C for 30 min. Then PDO in polymerized Matrigel were transferred into the round hole of a cassette and subjected to staining procedures. The primary antibodies used included CA‐9 (GeneTech, GT224004), PAX8 (GeneTech, GT210204), and E‐Cadherin (GeneTech, GT234807).

### Construction of Cell‐Derived Xenograft (CDX) Model

To construct the CDX model, 1 × 10^6^ 786‐O cells suspended in 100 µL PBS were subcutaneously injected into the right flank of 6‐week‐old female BALB/c nude mice (*n* = 6 per group). Mice in the PS‐MPs group were given drinking water containing 1‐µm PS‐MPs (10 mg L^−1^) starting 12 weeks prior to the experiment and continuing until the study conclusion. Mice in the BAY 11‐7082 group received daily intraperitoneal injection of BAY 11‐7082 at 20 mg kg^−1^, while those in the SB431542 group received daily intraperitoneal injection of SB431542 at 15 mg kg^−1^. Mice were euthanized by CO_2_ asphyxiation on day 27, and xenograft tumors were dissected for subsequent analysis. Tumor volume was calculated using the formula: tumor volume = 0.5 × length × width × width. The maximum tumor diameter was maintained within the IACUC‐approved limit of 2 cm.

### Statistical Analysis

The statistical tests applied were shown in the figure legends. All data were presented as mean ± SD unless otherwise stated. Statistical analysis was performed using GraphPad Prism software (Version 9.0). The difference was significant when *p* < 0.05.

## Conflict of Interest

The authors declare no conflict of Interest.

## Author Contributions

S.Y., J.X., S.Z., Q.G., and A.A. contributed equally to this work. S.Y. and W.X. conceived the conceptualization. W.X., H.Z., T.W., D.X., and D.Y. acquired resources. S.Y., J.X., S.Z., Q.G., and A.A. performed the data curation. K.C., G.W., J.L., and X.T. developed software. S.Z., Y.J., W.Z., T.C., and M.X. performed the formal analysis. W.X., H.Z., T.W., D.X., and D.Y. performed the supervision. S.Y., X.T., W.X., D.Y., and H.Z. performed the funding acquisition. S.Y., J.X., and S.Z. performed the validation. Q.G. and A.A. performed the investigation. S.Y., J.X., and S.Z. performed the visualization. S.Y. and W.X. performed the methodology. S.Y. and W.X. performed the writing of the original draft. W.X. and H.Z. performed the project administration. H.Z., T.W., D.X., and D.Y. performed the writing‐review and editing.

## Supporting information



Supporting Information

Supporting Information

## Data Availability

The data that support the findings of this study are available in the supplementary material of this article.
