# Supplementary material for: Polystyrene Microplastics Exposure Aggravates Clear Cell Renal Cell Carcinoma Progression via the NF‐κB and TGF‐β Signaling Pathways
Source: Adv Sci (Weinh). 2025 Nov 27;13(8):e18500. doi: 10.1002/advs.202518500 (PMC12884784; doi:10.1002/advs.202518500)
Supplement: Supplementary file 1 — Supporting Information [file ADVS-13-e18500-s001.docx]

**Figure S1. PS-MPs, BAY 11-7082, and SB431542 do not produce obvious hepatotoxicity and nephrotoxicity.**

**A, H** Body weight of mice with indicated treatment. **B, I** AST of mice with indicated treatment. **C, J** ALT of mice with indicated treatment. **D, K** CREA of mice with indicated treatment. **E, L** UREA of mice with indicated treatment. **F, M** Representative H&E staining for livers of mice with indicated treatment. Scale bar is 100 µm. **G, N** Representative H&E staining for kidneys of mice with indicated treatment. Scale bar is 100 µm. Statistical significance was determined by two-tailed unpaired t-test (A-E and H-L). * *P* < 0.05, ***P* < 0.01, *** *P* < 0.001, **** *P* < 0.0001, and ns *P* ≥ 0.05. Experiments were independently repeated three times with similar results; data of one representative experiment are shown (F, G, M, and N).
